# Challenges and potentials of new breeding techniques in *Cannabis sativa*

**DOI:** 10.3389/fpls.2023.1154332

**Published:** 2023-06-08

**Authors:** Christina Rønn Ingvardsen, Henrik Brinch-Pedersen

**Affiliations:** Crop Genetics and Biotechnology, Department of Agroecology, Aarhus University, Slagelse, Denmark

**Keywords:** *Cannabis sativa*, medical Cannabis, drug type Cannabis, genome editing, plant regeneration, transformation

## Abstract

*Cannabis sativa* L. is an ancient crop used for fiber and seed production and not least for its content of cannabinoids used for medicine and as an intoxicant drug. Due to the psychedelic effect of one of the compounds, tetrahydrocannabinol (THC), many countries had regulations or bands on Cannabis growing, also as fiber or seed crop. Recently, as many of these regulations are getting less tight, the interest for the many uses of this crop is increasing. Cannabis is dioecious and highly heterogenic, making traditional breeding costly and time consuming. Further, it might be difficult to introduce new traits without changing the cannabinoid profile. Genome editing using new breeding techniques might solve these problems. The successful use of genome editing requires sequence information on suitable target genes, a genome editing tool to be introduced into plant tissue and the ability to regenerate plants from transformed cells. This review summarizes the current status of Cannabis breeding, uncovers potentials and challenges of Cannabis in an era of new breeding techniques and finally suggests future focus areas that may help to improve our overall understanding of Cannabis and realize the potentials of the plant.

## Introduction to medical Cannabis

Cannabis (*Cannabis sativa* L.) is an annual, predominantly dioecious (male and female flowers occur on separate plants), rarely monoecious (male and female flowers occur on the same plant) plant (Chandra et al., 2020). It is wind pollinated and flowers under short day conditions. Cannabis has a diploid genome (2n = 20) and its karyotype is composed of 9 pairs of autosomes and one pair of sexual chromosomes (X and Y) (Divashuk et al., 2014). The plants have been used by man for at least 6000 years (Li, 1974). The existence of an extant natural population is doubtful, and any ‘wild’ species might be naturalized from domesticated plants indicating that the current genetic variation is most probably due to the action of man (Small, 2017). Cannabis has traditionally been classified as a single species (Small and Cronquist, 1976). This is supported by molecular studies (Oh et al., 2016; Zhang et al., 2018). However, Cannabis is often divided into subspecies or groups based on chemotype, ecotype, crop-type (fiber or drug) or leaflet morphology (Grassi and McPartland, 2017; McPartland, 2017; Small, 2017). Grouping is often problematic, as the types readily inter-cross and a lot of hybrids exist and as classification based on crop-type is somewhat dependent on legislation. In this paper, we focus on Cannabis used for medical purposes, e.g., plants with a high level of cannabinoids, and will therefore use the term hemp for Cannabis grown for fiber and seeds and the term medical or drug type Cannabis for varieties grown for their cannabinoid content, independent of the tetrahydrocannabinol (THC)/cannabidiol (CBD) ratio.

Cannabis produces a range of secondary metabolites, the best known are the phytocannabinoids. Although as many as 120 different cannabinoids have been reported (see Radwan et al. (2017) for review), the most abundant cannabinoids are THC, CBD, cannabigerol (CBG) and cannabinol (CBN) (Flores-Sanchez and Verpoorte, 2008; Fischedick et al., 2010; ElSohly et al., 2016). Tetrahydrocannabinolic acid (THCA) and cannabidiolic acid (CBDA) are made from the common precursor, cannabigerolic acid (CBGA) whereas CBN is an oxidation product of THC. Acidic forms of cannabinoids are biosynthesized in the trichomes on the female flowers (Sirikantaramas et al., 2005; Livingston et al., 2020). The storage of the toxic cannabinoids in trichomes minimizes the risk of self-intoxication (Sirikantaramas et al., 2005). The acidic forms are spontaneously decarboxylated into THC and CBD by heat and light. THC is the main psychoactive compound in drug type Cannabis (Pertwee, 1988), but also beneficial effects are reported (Grotenhermen, 2003). Moreover, other cannabinoids, especially CBD, have attracted interest for their pharmacological properties. CBD has been reported to act as an antidepressant, to relieve pain and anxiety and to reduce inflammation. It is believed to have beneficial effects in a variety of diseases, such as Alzheimer’s and Parkinson’s diseases, multiple sclerosis, Huntington’s disease, epilepsy, and cancer (Pisanti et al., 2017). The resin from the glandular trichomes also contain terpenes (Flores-Sanchez and Verpoorte, 2008). Terpenes does not only add flavor to the product but also have medical properties, not least in synergic action (entourage effect) with cannabinoids (Ferber et al., 2020).

*In planta*, the secondary metabolites are believed to protect the plants against various pathogens and insects. Both cannabinoids and terpenes have been shown to possess antifungal activity (McPartland, 1984; Wanas et al., 2016) and to be toxic to insects (Taura et al., 2007; Mithöfer and Boland, 2012; Bedini et al., 2016). Although the active component is not known, Cannabis resin might have antibacterial activity (Radošević et al., 1962) and extract of Cannabis leaves is active against nematodes (Mukhtar et al., 2013).

Solving the many challenges in Cannabis sativa calls for the use of all available tools. In the current review, we discuss the challenges and possibilities for New Breeding Techniques, such as genome editing in medical Cannabis. To set the scene, we first present a brief status of Cannabis breeding and the level of genetic diversity. We then address the requirements for successful tissue culture and transformation with a focus on the status of micro propagation, regeneration and transient as well as stable transformation. Finally, we discuss the many opportunities for using genome editing in the improvement of medical Cannabis.

## Breeding, genetic diversity and genetic markers

### Breeding

Due to the high-value products, medical Cannabis is often produced in greenhouse or indoor facilities where the plants are propagated like a horticultural crop using stem cuttings (Vassilevska-Ivanova, 2019; Monthony et al., 2021c). The use of cuttings ensures that only female plants with a higher level of cannabinoids are used for production. The dioecy behavior of Cannabis, making it an obligately outbreeding species, the limited number of genetic markers and the anecdotal start of breeding of medical cultivars make breeding of drug type Cannabis challenging. Early breeding was selection, done by the illegal market with decades of interbreeding and hybridization without record of parentage (Barcaccia et al., 2020; Gilchrist et al., 2023). This means that the genetic identity of a medical Cannabis strains cannot be reliably inferred from its name, as studies have shown that some strains with different names were genetically similar, and some strains with identical names were genetically different (Sawler et al., 2015; Dufresnes et al., 2017; Punja et al., 2017; Reimann-Philipp et al., 2020; Adhikary et al., 2021). However, the fact that breeding has been illegal does not mean that it is inefficient, as the level of THC has increased (Mehmedic et al., 2010). For a comprehensive review on medical Cannabis breeding, readers are referred to Barcaccia et al. (2020).

The level and composition of cannabinoids and terpenoids as well as stability in production, flowering time and lower resource input are in focus in modern medical Cannabis breeding, with more focus on CBD and other non-psychoactive cannabinoids. Resistance against insects, pathogens and viruses is also in high demand. Healthy mother plants are essential. However, maintenance of mother plants in contained humid environments poses a challenge in relation to attacks by plant pathogens such as powdery mildew. This significant challenge can only be kept at tolerable levels by a strict growth control including air circulation, ventilation, and moisture control as the strict regulations for medical products do not permit any use of pesticides.

### Doubled haploids

The production of doubled haploids (DH) in Cannabis would be highly advantageous, as it would be possible to produce female pure lines in one generation. Haploid plants have in other plant species been produced *via* androgenesis (anther or microspore culture), gynogenesis, parthenogenesis or wide hybridization-chromosome elimination (Ishii et al, 2016; Hooghvorst and Nogués, 2021). Later chromosome duplication in the haploid plants is performed, either spontaneously or by chemical treatment. Although DH production *via* microspore culture has been investigated in Cannabis, successful DH production has so far not been established (Adhikary et al., 2021). Cannabis seems to be recalcitrant to androgenesis induction, although very few embryos can be developed (Galán-Ávila et al., 2021a). The method used for successful doubled haploid production seems to be species dependent wherefor also the other methods should be investigated for their usefulness in Cannabis. Recently, CRISPR/Cas have been used to develop haploid-inducer lines in both monocot and dicot plants (Kelliher et al., 2019). As also suggested by others (Hesami et al., 2021a; Simiyu et al., 2022), this method might be very useful in Cannabis.

### Polyploidization

Polyploidization is used as a tool in plant breeding to improve desirable plant characteristics such as larger organs and higher yield (Sattler et al., 2016). Even though the expected “giga” effect is not always achieved, higher production of secondary metabolites in autotetraploid medical plants is seen in several cases as reviewed by others (Dhawan and Lavania, 1996; Iannicelli et al., 2020; Niazian and Nalousi, 2020). As genomic stress occurs after polyploidization, genomic rearrangements, gene loss and/or changes in gene expression might occur (Iannicelli et al., 2020; Niazian and Nalousi, 2020). This might give a changed chemical profile with lower or even missing production of secondary metabolites, making it difficult to predict the outcome of polyploidization in new species or even other genotypes.

In Cannabis, the effect of polyploidization has been studied in hemp as well as drug-type Cannabis (Bagheri and Mansouri, 2015; Mansouri and Bagheri, 2017; Parsons et al., 2019; Hesami et al., 2021a). In both types, the tetraploid plants had broader leaves with bigger and less dense stomata, both clear signs of polyploidization. Cuttings of the drug-type Cannabis had reduced rooting ability, a phenomenon also observed in hop (Trojak-Goluch and Skomra, 2013; Parsons et al., 2019). When the level of cannabinoids was analyzed, only small changes were found. Levels of CBD was increased by 9%, whereas the level of THC was unchanged in drug-type Cannabis (Parsons et al., 2019). In the hemp-type plants, the level of THC was reduced in the female flowers with no change in the amount of CBD (Bagheri and Mansouri, 2015).

The terpene profile was not analyzed in the hemp-type Cannabis, but in the drug-type, the terpene profile changed after polyploidization, as mainly the contents of sesquiterpenes increased (Parsons et al., 2019). A change in terpene profile after polyploidization was also found in hop. Although the general level was lower, there was an increase in terpenes desirable for the brewing industry (Trojak-Goluch and Skomra, 2013).

These experiments did not show very promising results as far as an increase in cannabinoid is concerned. It should be reminded, however, that only one genotype per experiment gave tetraploid plants that could be analyzed. As there is often a difference between genotypes, the effect of polyploidization on the level of cannabinoids, terpenes and other important traits might have a different and more positive outcome in other trials, not least after crossing of polyploids with different genetic backgrounds.

### Genetic structure and diversity

Several studies using STRUCTURE analysis position hemp and drug type Cannabis in separate clusters (Sawler et al., 2015; Lynch et al., 2016; Dufresnes et al., 2017). The genetic differences between the groups are distributed across the genome and are not restricted to loci involved in cannabinoid production (Dufresnes et al., 2017b; Sawler et al., 2015). Whether hemp or drug type Cannabis is having more heterozygosity seems to depend on the study, which probably reflects differences in the selected cultivars (Sawler et al., 2015; Lynch et al., 2016). The lower genetic diversity in drug type Cannabis compared to hemp found in some studies (Sawler et al., 2015) might be due to inbreeding and genetic bottlenecks in the illegal Cannabis production. However, studies including a broader set of genotypes are needed to create more knowledge about the heterozygosity of Cannabis from all regions (Kovalchuk et al., 2020). Such studies also provide the widest genetic background for medical Cannabis breeding.

Genetic markers such as single sequence repeats (SSRs) and Inter Simple Sequence Repeats (ISSR) have shown a high degree of genetic diversity in drug type Cannabis (Punja et al., 2017; Soler et al., 2017; de Oliveira Pereira Ribeiro et al., 2020). The analyses not only showed diversity between but also within cultivars (Punja et al., 2017; Soler et al., 2017). The high diversity within a cultivar means that hardly any reduction in the genetic variation was found after one round of selfing (Punja and Holmes, 2020). Some of this high variation found after selfing might be due to accumulation of somatic mutations within plants been propagated as cuttings for a long time (Adamek et al., 2022).

The high genetic diversity found in Cannabis is very useful for breeding new varieties. For medical Cannabis, however, homogenous material is needed, and varieties must be multiplied *via* cuttings. Introduction of single gene traits like disease resistance genes in Cannabis by traditional cross breeding, without affecting the genetic background and thereby the cannabinoid and terpene profile is difficult. Genome editing might solve this issue, see below.

### Use of genetic markers

The use of genetic markers in drug-type Cannabis has mainly focused on analysis of Cannabis samples and plants to discriminate between hemp type and drug type material, to evaluate genetic variance and to identify female plants. Some examples will be highlighted here, for more comprehensive information, especially on early work, readers are referred to Onofri and Mandolino (2017); Punja et al. (2017) and Hesami et al., 2020. Further, a panel of 41 robust SSRs, with an average of four markers per chromosome, is provided by Barcaccia et al. (2020).

#### Markers for chemotype

As drug type Cannabis is illegal in many countries, there is a great need to be able to detect the presence of this type of Cannabis in seized samples. An important issue to consider here is the balance between speed, simplicity of the analysis, affordability, and accuracy. Many different assays have been developed, not only to discriminate between hemp and drug types, but also to establish from which geographic location the sample might originate.

The simplest form of markers is based on the genes for tetrahydrocannabinolic acid synthase (THCAS) and cannabidiolic acid synthase (CBDAS). As DNA extracted from the seized material is analyzed, there is no need to wait for plants to grow in case of seed material or to extract cannabinoids to analyze for the level of THC and CBD. The markers reported by Kojoma et al. (2006) and Rotherham and Harbison (2011) was based on THCAS, only. At the time, it was believed that the two enzymes belonged to the same locus so that plants having the THCA synthase would contain only THC or a mix of THC and CBD. Other markers are based on both THCAS and CBDAS sequences (Weiblen et al., 2015; Welling et al., 2016; Cascini et al., 2019; Toth et al., 2020), which is in line with newer information showing that the two enzymes are found in two closely linked loci (Laverty et al., 2019).

Other types of markers have also been shown to be useful; autosomal microsatellite markers and markers based on mitochondrial and chloroplast DNA. SSR’s have been used to differentiate between samples (Soler et al., 2016; Dufresnes et al., 2017; Houston et al., 2017; Soler et al., 2017; Houston et al., 2018; de Oliveira Pereira Ribeiro et al., 2020; Ioannidis et al., 2022b). It seems that a limited number of markers, from 6 to 13, is enough, not only to sort Cannabis from hop, but to individualize and differentiate between types (drug versus hemp) and to say something about geographic origin (Houston et al., 2017; de Oliveira Pereira Ribeiro et al., 2020). Often markers can be multiplexed (Dufresnes et al., 2017; Houston et al., 2017; de Oliveira Pereira Ribeiro et al., 2020), reducing labor and cost.

Thus, genetic markers are useful as forensic tool to give a confirmation whether a sample is Cannabis and to discriminate between hemp and drug type samples. This can otherwise be difficult, especially with seed samples where there is no obvious difference. Also, information about the geographical origin of samples can give valuable information about distribution routes of illegal products.

#### Sex markers

A mentioned, the karyotype is composed of 9 pairs of autosomes and one pair of sexual chromosomes (X and Y) (Divashuk et al., 2014). Sex determination in dioecious Cannabis is believed to function through a X-to-autosome balance system, where X/A = 1 are female (XX) and X/A = 0.5 are male (XY) rather than by a Y-active system (Ainsworth, 2000; Vyskot and Hobza, 2004). Monoecious hemp cultivars having both male and female flowers on the same plant, have two X chromosomes (Faux et al., 2014; Razumova et al., 2016). Different genetic markers have been developed to sort male and female plants (Mandolino et al., 1999; Mandolino et al., 2002; Törjék et al., 2002; Toth et al., 2020). It seems that the sex determination is somewhat leaky, as the environment, especially photoperiod, hormones and unknown genetic components other than the sex chromosomes also plays a role (Schaffner, 1921; Faux et al., 2014). In female drug-type Cannabis, spontaneous formation of hermaphroditic plants, with both female and male flowers, can be as high as 10% (Punja and Holmes, 2020). This ability of female plants to produce male flowers independent of the presence of the Y chromosome is used in the production of feminized seeds, as female plants can be treated with thiosulfate to produce male flowers (Lubell and Brand, 2018).

## The prerequisites for genome editing

Due to the many uses of Cannabis, genome editing would be a very desirable tool. Not only would we get a deeper insight into the cannabinoid pathway, but also many other genes important for the many uses of Cannabis could be investigated. The successful use of genome editing requires a genome editing tool, sequence information of suitable target genes, introduction of the construct into plant tissue and the ability to regenerate shoots from explant tissue.

### Gene editing techniques

New Breeding Techniques (NBT) have emerged as alternatives to classical plant breeding and conventional transgenesis. These new techniques facilitate development of novel varieties more precisely and faster than by classical breeding giving genome modifications indistinguishable from those introduced by conventional breeding and chemical or physical mutagenesis (Lusser et al., 2012). NBT include the sequence-specific nuclease (SSN) tools such as Zinc Finger Nucleases (ZNF´s) (Petolino, 2015), Transcription Activator-Like Effector Nucleases (TALENs) (Khan et al., 2017) and Clustered Regularly Interspaced Short Palindromic Repeats (CRISPR) (Chen et al., 2019; Montecillo et al., 2020), all allowing for targeted mutagenesis of candidates without unwanted side mutations.

A common feature for ZFNs, TALENs and CRISPR/Cas is the programmability to cleave in specific locations and generate DNA double strand breaks (DSBs) that stimulate standard cellular repair mechanisms including non-homologous end joining (NHJE) and homology-directed repair (HDR) (Voytas, 2013; Gupta et al., 2019). By NHEJ, the repair at the DSB site is often imprecise which leads to introduction of small deletions/insertions at the site of break, resulting in knockout of gene function *via* frame shift mutations. HDR requires a homologous DNA segment as template to correct or replace existing sequence. By HDR it is thus possible to make specific nucleotide changes.

ZNF´s and TALENs are hybrid proteins created by fusing ZF and TALE DNA-binding domain to the non-specific cleavage domain of FokI endonuclease (Petolino, 2015; Khan et al., 2017). The FokI endonuclease non-specific cleavage domain must dimerize to cleave the DNA target. In August 2013, CRISPR/Cas emerged as an alternative genome editing method in plants (Feng et al., 2013; Li et al., 2013; Nekrasov et al., 2013; Shan et al., 2013; Xie and Yang, 2013). The CRISPR method was originally discovered as an antiviral defense system widespread in prokaryotes (Wiedenheft et al., 2012). The system is based on a Cas9 nuclease which can be targeted to a specific genomic sequence by an easily engineered single guide RNA (sgRNA) of 20 base pair (bp). A protospacer adjacent motif (PAM) is needed adjacent to the 3’ end of the 20 bp target. Originally, the purpose of the PAM sequence was to distinguish self from non-self in prokaryotes.

The CRISPR/Cas technology has become the preferred method for making targeted mutations in plants without undesired side mutations, due to ease of use, precision, efficiency, and low cost (Shan et al., 2013; Kumar et al., 2020), and since first reported a long range of rapports on modifications in crops have been reported in multiple plant species (Gupta et al., 2019). Ease of multiplexing, i.e., the simultaneous targeting if several genes with a single molecular construct, is another major advantage of CRISPR/Cas9 technology compared to ZFN and TALEN (Shan et al., 2013; Lowder et al., 2016; Ma et al., 2016; Čermák et al., 2017; Kumar et al., 2020). Although many report the use of CRISPR techniques in the coding sequence of genes, they can be used in promoters and upstream open reading frames as well (Holme et al., 2017; Rodríguez-Leal et al., 2017; Si et al., 2020). Recently DNA editing in plant plastics has also become a possibility (Kang et al., 2021).

The technology is quickly developing (Zhu et al., 2020), and new CRISPR nucleases like Cas12a creating 5´overhangs or nucleases having different PAM recognition sites are constantly expanding the perspective (Jaganathan et al., 2018; Swarts and Jinek, 2018). Very recently, PAM-less CRISPR systems are emerging (Ren et al., 2021). Nucleases that only make a single-strand break (nickases) can be used in pairs, each requiring a sgRNA. By positioning the two nicks close to each other on opposite strands, a break is created. As two sgRNAs are required, this paired nicking dramatically increases the specificity and thus reduces off-targeting in unwanted places. HDR is still challenging in plants and several developments in the CRISPR techniques have been developed to overcome this problem. Adenine base editors (ABEs) changing adenine (A) to guanine (G) and cytosine base editors (CBEs) changing cytosine (C) to thymine (T) are now working in plants (Zong et al., 2018; Hua et al., 2020). A new technique for editing is the prime editing technique, where bases in the target site is edited based on the sequence of a prime editing guide RNA (Lin et al., 2020; Zhu et al., 2020; Zong et al., 2022). Further, multifunctional genome editing systems are now emerging, making it possible to do both base editing and knockout simultaneous using a single construct (Li et al., 2020). On top of local changes such as minor deletions/insertions and base editing, the introduction of bigger structural variations are now also possible (Lu et al., 2021). Some new techniques are firstly developed for monocots and optimization might be needed to use them in dicots such as Cannabis.

Online tools are available to guide the design of efficient sgRNAs. Five different sgRNA designing tools and their main characteristics have been reviewed by Hesami et al. (2021a). The algorithms in such programs are developed based on the assessment of many thousands of gRNAs targeting genes. However, most tools are not developed based on plant data and the predicted efficiency is not always in accordance with the found results (Pauwels et al., 2018; Naim et al., 2020). An updated tool based on plant data is needed to increase the efficiency of plant sgRNAs.

### Status of available sequence data

There is now a reference genome for Cannabis, which can be found at NCBI (**Supplementary Table S1**). This reference genome, called cs10, is made from female material in the CBD-high isolate CBDDRx-18 (Grassa et al., 2021). On top of the reference genome, NCBI lists thirteen different Genbank accessions of genome assemblies, five of which are at chromosome level (**Supplementary Table S1**). These thirteen assemblies mostly represent female plants but are made from different types of Cannabis (TDC high, CBD high, mixed profile and hemp), making them a valuable tool for finding sequence data for potential candidate genes. As Cannabis is highly heterozygous, not least at the THCAS and CBCAS gene loci, the creation of a Cannabis pangenome is in high demand (Hurgobin et al., 2021). Six different transcriptome assemblies from hemp and medical Cannabis are also available from NCBI and the Cannabis Genome Browser (**Supplementary Table S2**). On top of this, very many sequences (Nucleotide, WGS, RNA-seq, Nanopore and Targeted amplicon sequences form the Phylos Bioscience/Open Cannabis Project) can be blasted from the NCBI homepage, if the gene of interest should not appear in the genome assemblies. Reference genomes and other assemblies for both the chloroplast and mitochondrial genomes are also available (**Supplementary Table S3**).

It must be stressed that for gene editing techniques, the precise genetic sequence of the cultivar at hand is needed in order to design guides. This means that both alleles of each candidate gene to be targeted must be sequenced. Further, the availability of genomic sequence information not only provide the candidate genes for targeting but also gives the opportunity to analyze for possible off-targets, not only in gene families with high sequence similarity but also unexpected off-targets in unrelated genome regions. However, a very accurate screening for off-targets might be difficult in the highly heterozygous Cannabis genome.

### Tissue culture and plant regeneration

#### Micropropagation

The plant material for production of medical Cannabis is obtained from cuttings from mother plants. The maintenance of mother plants demands a lot of space and considerable effort to keep the material free from diseases. Further, there is a need to store valuable breeding material. Propagation and maintenance of material *via in vitro* culture would be a way to solve this issue and tissue culture has been a topic in several papers. Several papers have reported methods for *in vitro* propagation in Cannabis, methods that often are described as regeneration (**Table 1**) (Lata et al., 2016; Piunno et al., 2019; Smýkalová et al., 2019; Ioannidis et al., 2020; Wróbel et al., 2022). However, most papers do not describe regeneration from a single non-differentiated cell but instead report shoot formation from pre-existing meristems, once the apical dominance is broken. Summaries of published micropropagation studies were recently published (Hesami et al., 2021a; Monthony et al., 2021c). Readers with special interest in micro propagation and/or preservation are referred to these papers.

**Table 1:** List of *Cannabis sativa* studies reporting micropropagation from pre-existing meristems.

Type of explant	Genotype (Type)	Number of shoots obtained	References
Nodal segments	MX-1 (drug)	13.8 shoots/explant	Lata et al., 2009a
Shoot tips	Changtu (fiber)	3.22 shoots/shoot tip	Wang et al., 2009
Nodal segments	Mexican variety (drug, THC rich)	13.44 shoots/explant	Lata et al., 2016
Immature and mature floral explants	1KG2TF (drug, THC rich)	4^a^ shoots/immature inflorescence	Piunno et al., 2019
	S1525 (drug, THC rich)	< 1^a^ shoot/immature inflorescence	
Isolated meristem	USO-31 (fiber, < 3% CBD)	4.4 shoots/explant	Smýkalová et al., 2019
Shoot apex		3.2 shoots/explant	
Cotyledonary node		2.4 shoots/explant	
Nodal segments	High CBD variety	3.63 shoots/explant	Ioannidis et al., 2020
	High CBG variety	3.38 shoots/explant	
Nodal segments	MX-CBD-11 (drug, CBD rich)	3.00 shoots/explant	Mestinšek-Mubi et al., 2020
	MX-CBD-707 (drug, CBD rich)	2.43 shoots/explant	
Shoot tips of epicotyl	Diana	3.4 shoots/explant	Dreger and Szalata, 2021
	Fedora 17	3.2 shoots/explant	
	Finola (all hemp)	3.7 shoots/explant	
2 cm stem tips	Wife (hemp)	8.4 micro-cuttings/4 explants	Lubell-Brand et al., 2021
Meristems	Pure CBD	1.9 shoots/explant	Holmes et al., 2021
Nodal segments	Cheesequake	0.5 shoots/explant	
	Moby Dick	1.9 shoots/explant	
	Space Queen	0.2 shoots/explant	
	Pennywise (all drug type)	2.0 shoots/explant	
	BLD	0.4 shoots/explant	
	SWD	0.4 shoots/explant	
	Moby Dick (all drug type)	0.3 shoots/explant	
Single floret	U82 (drug, THC rich)	18.2 shoots/explant	Monthony et al., 2021a
Pair of florets		14.7 shoots/explant	
Shoot tip	US Nursery Cherry I (drug)	5.75 shoots/explant, over 4 cycles	Murphy and Adelberg, 2021
Two-node explants	BA-21 (drug, code name)	2.23 shoots/explant	Page et al., 2021
	BA-41 (drug, code name)		
	BA-49 (drug, code name)		
	BA-61 (drug, code name)		
	BA-71 (drug, code name)		
Tip cuttings	Epsilon 68 (fiber, CBD rich)	2.5 shoots/explant	Wróbel et al., 2022
Nodal cuttings		1.3 shoot/explant	
Secondary tip and nodal cuttings		3 shoots/explant	
Microshoots	Abacus (hemp)	2.6 shoots/explant	Borbas et al., 2023
	Wife (hemp)	3.7 shoots/explant	
Stem segments with two nodes	Honey Banana (THC-rich)	1.5 shoot/explant	Hesami et al., 2023
1-cm single-node stem segment	TJ’s CBD (CBD-rich)	>20^a^ shoots/explant (TDZ, low quality)	Stephen et al., 2023
		8^a^ shoots/explant (BA, high quality)	
Shoot segments with one node	Safari Punch (THC-dominant)	5.33 shoots/explant	Zarei et al., 2023
	Peanut Sundae (THC-dominant)	2^a^ shoots/explant	
	Master Hemp (CBD-dominant)	5.4 shoots/explant	
	Orange Mimosa Purple (THC rich)	2.4^a^ shoots/explant	
	Golden Papaya (THC-dominant)	3.33 shoots/explant	

Only genotypes from which shoots were obtained are included. ^a^ number estimated from figure.

In general, finding the right growth medium seems to be very cultivar dependent (Grulichova et al., 2017; Codesido et al., 2020; Stephen et al., 2023). This makes it difficult to compare studies using different combinations of cultivars and media. It remains to be uncovered if this is due to a very limited number of plants being very willing to respond *in vitro*, or whether the ideal medium composition still needs to be discovered. This cultivar dependence implies that the use of hemp as a proxy for medicinal type Cannabis may not be successful (Page et al., 2021). Although there might not be a big difference in multiplication rate between fiber and drug types (**Table 1**). The results might also be dependent on whether the starting material is taken directly from the greenhouse or from tissue that has been in tissue culture for some time. The position (basal versus apical) of the stem explant also seems to affect multiplication rates (Hesami et al., 2023). There could be a lingering effect from plant growth regulators found in plants with roots that might influence the multiplication rate (Page et al., 2021). A high number of shoot proliferation has been reported in some cases (Lata et al., 2009a; Lata et al., 2016) but in most cases the number of shoots per explant is only between 2 and 3 (**Table 1**). The use of floral reversion might be a way to move forward, as this method increased the multiplication rate up to eightfold (Piunno et al., 2019; Monthony et al., 2021a). It should be noted that achieving the highest multiplication rate might give lower quality and lower rooting ability (Stephen et al., 2023). Rooting is very important for successful micropropagation and has been a topic in several papers (e.g.: Zarei et al., 2021; Ioannidis et al., 2022a; Kurtz et al., 2022; Stephen et al., 2023).

There is a general need for improvement in more cultivars, if micropropagation should be of general use. Scientific research can be successful, even though a very limited (or only one) number of plant cultivars can be used. However, in production, medical Cannabis growers need to be able to use the technique in all their material before they invest in tissue culture facilities. It is most probable that several companies already have developed successful protocols for micropropagation. Most of these are, however, kept as trade secrets, if not funded by public means (Adhikary et al., 2021; Zarei et al., 2023). Resent papers show that improvement of micropropagation is still an interest also in universities (Borbas et al., 2023; Hesami et al., 2023; Stephen et al., 2023) and new methods, such as the use of bioreactors (Rico et al., 2022), photosynthetic proficiency measurements (Pepe et al., 2022) and photoautotrophic micropropagation (Zarei et al., 2021) have been investigated.

Micropropagation using synthetic seeds has been investigated as an alternative solution for propagation and conservation of germplasm. The use of synthetic seeds in Cannabis was first reported by Lata et al. (2009b; Lata et al., 2012) using the cultivar MX. Recently, a paper on commercial scale synthetic seed production using the elite cultivar ‘Slurricane’ was published (Zarei et al., 2022). The developed method was very successful, as a regrowth rate of 100% was seen after storage for 150 days. Cryopreservation has also been investigated as a means for long term storage on *in vitro* material (Lata et al., 2019; Downey et al., 2021). Although these methods are not of immediate importance for genome editing, they represent valuable ways of storing high-value material such as modified cultivars.

#### Plant regeneration

*In vitro* propagation from pre-existing meristems is very useful for multiplying material for medical Cannabis production. However, if regeneration from single cells is optimized, a much higher multiplication rate might be obtained. Further, to produce CRISPR/Cas mutated plants, an efficient protocol for *de novo* regeneration of plants from single cells is needed to avoid chimeric plants. In Cannabis, such *de novo* regeneration seems very difficult to obtain from callus or tissue without preformed meristems. This recalcitrance to regeneration is the main obstacle to an efficient genome editing protocol in Cannabis (Monthony et al., 2021b).

Recalcitrance is a common problem in tissue culture (Altpeter et al., 2016). For years, many different combinations of explants and plant growth regulators have been used to try to solve the difficulty of regenerating plants from very many species, often without great success. The ability to regenerate seems to be not only species dependent but also cultivar dependent. The recalcitrance might be linked to a very high degree of apical dominance and/or difficulties in cellular reprogramming of already differentiated cells (Sugimoto et al., 2019), making the generation of shoots difficult. This calls for further research into whether there is a general explanation for recalcitrance to regenerate across plant species.

Testing many different combinations of explant type, explant age, type of gelling agent, type of carbohydrate source, type, and balance of PGRs and addition of other supplements such as Zn or polyamines is often tedious and time consuming, sometimes with a low success rate. This process might be optimized using the ability of machine learning to discover non-linear relationships and concealed interactions (García-Pérez et al., 2020). Machine learning has already been used in Cannabis to optimize *in vitro* seed germination (Hesami et al., 2021b; Pepe et al., 2021a; Aasim et al., 2022) and to study *in vitro* shoot growth and development (Pepe et al., 2021b) and callus morphology (Hesami and Jones, 2021). Further, as many research projects are performed by PhD students and post docs, with a strong demand for an outcome of scientific papers, the focus is often turned away from comprehensive, long-term research aiming at optimizing regeneration protocols. There might also be a lack of reports showing negative results. All this might have slowed down the progress of regeneration.

A direct comparison between experiments is sometimes difficult as results are given as responding explant % or number of shoots per shooting explant (**Table 2**). Although the lowest percentage of responding explants is reported in hemp (Slusarkiewicz-Jarzina et al., 2005), there is not a clear trend for a difference between hemp and drug type cultivars. The biggest difference can be explained by the type of explants used. Experiments using hypocotyl, stem, or stem nodes have a higher response rate in general (**Table 2**) (Wielgus et al., 2008; Galán-Ávila et al., 2020; Önol and Yildirim, 2021; Galán-Ávila et al., 2021b).

**Table 2:** List of *Cannabis sativa* studies reporting regeneration experiments without transformation.

Type of explant	Genotype	Type	Callus stage	Best outcome		References
				Responding explants (%)	No. of shoots per explant	
Axillary bud Petiole Internode	Fibrimon-24, Silesia, Novosadska, Fedrina-74 Juso-15	Hemp Hemp Hemp	Yes Yes Yes	2.3 2.5 1.5		Slusarkiewicz-Jarzina et al., 2005
Stem Cotyledon	Bialobrzeskie, Beniko, Silesia Bialobrzeskie, Beniko, Silesia	Hemp Hemp	Yes Yes	14 6		Wielgus et al., 2008
Young leaf	MX	Drug	Yes	96.6	12.3	Lata et al., 2010
Epicotyl Cotyledon	Iranian Cannabis	Unknown	Yes Yes		2 1	Movahedi et al., 2015
Cotyledon	Kunming, Neimeng 700, YM535, Anhui727, DaliA1, Heilongjiang698, Heilongjiang449 BM2	Hemp Hemp Seed	Yes	54.8	3.0	Chaohua et al., 2016
Hypocotyl Cotyledons Leaf	Ferimon, Felina32, Fedora17, USO31, Finola Ferimon, Felina32, Fedora17, USO31, Finola Ferimon, Felina32, Fedora17, USO31, Finola	Hemp Hemp Hemp	No No No	71.15 9.29 average 0.42	1.72 1.42 -	Galán-Ávila et al., 2020
Hypocotyl Cotyledons	Ferimon, Felina32, Fedora17, USO31, Finola, Futura75 Ferimon, Felina32, Fedora17, USO31, Finola, Futura75	Hemp Hemp	No No	76.5 26.3	1.6 2.0	Galán-Ávila et al., 2021b
Leaf segments	UP305	Drug	Yes	18		Hesami and Jones, 2021
Stem nodes	Samsun Vezirköprü population	Drug	Yes	75	4.593	Önol and Yildirim, 2021
True leaves Cotyledon Hypocotyl Embryo hypocotyls of immature grains	YUNMA7 One hundred Cannabis varieties	Hemp Hemp	Yes Yes Yes Yes	2.27 1.78 1.41 7		Zhang et al., 2021
Stem	H-CBD variety H-CBG variety	Drug Drug	Yes Yes	62		Ioannidis et al., 2022b

One experiment, using leaf explants, stands out, as almost all explants gave shoots, with an average of 12.3 shoots per callus (Lata et al., 2010). This study has been replicated using ten other drug type Cannabis genotypes (Monthony et al., 2021b). Here, the experiment failed to induce shoots in all the genotypes tested, making it clear that regeneration might not only be tissue specific but also very dependent on genotype. The importance of genotype for regeneration in Cannabis is stressed by the fact that Zhang et al. found a regeneration rate varying from 0 to 7% when screening one hundred genotypes (Zhang et al., 2021). Further, the lack of reproducibility stresses the difficulty of transferring tissue culture methods to other laboratories.

Recently, Galán-Ávila et al. published two papers showing a very high percentage of direct regeneration without a callus phase from hypocotyls (**Table 2**) (Galán-Ávila et al., 2020; Galán-Ávila et al., 2021b). These results are very promising but remains to be seen whether this method can be transferred to medical Cannabis.

### Transformation

Transformation in Cannabis has been a research topic for more than 20 years. Three main transformation techniques have been used, the transformation with *Agrobacterium rhizogenes* to get hairy roots, transient transformation, and stable transformation. The status of transformation in Cannabis has also been reviewed by others (Feeney and Punja, 2017; Simiyu et al., 2022). For an in-depth discussion of Agrobacterium strains, promoters and selection markers, readers are referred to Hesami et al. (2021a).

Hairy root cultures often have an enhanced ability to synthesize secondary metabolites (Srivastava and Srivastava, 2007). Hairy root cultures were established after transformation with *A. rhizogenes* in hemp as well as drug-type Cannabis (Wahby et al., 2013; Wahby et al., 2017). Several types of media containing a range of different combinations of hormones were tried, but although callus developed from the hairy root cultures, no shoots were obtained (Wahby et al., 2017). No cannabinoids were present in the hairy root cultures. Similarly, no cannabinoid production was found in cell suspension cultures (Flores-Sanchez et al., 2009). Contrary, hairy root cultures developed from callus without the use of *A. rhizogenes* produced a very low level of cannabinoids (Farag and Kayser, 2015). The level of cannabinoids might be dependent on the variety of plant and/or of the plant variety-bacterial stain combination. However, higher levels would probably be toxic to the cultures. The ability of callus cultures to form roots was also seen by Feeney and Punja (2003). Unfortunately, regeneration of plants from hairy roots, although possible in some species (Crane et al., 2006; Lütken et al., 2012; de la Torre et al., 2018), is often quite challenging and has not yet been reported in Cannabis.

Transformation using *Agrobacterium tumefaciens* is often used in plants, both for transient and stable transformation (Dunwell and Wetten, 2012; Krenek et al., 2015). Studies with wild-type *A. tumefaciens* (MacKinnon et al., 2001; Wahby et al., 2013) as well as recent papers (**Tables 3**, **4**) (Galán-Ávila et al., 2021b; Zhang et al., 2021) show that genetic transformation of Cannabis is possible. However, there seems to be a cultivar difference in the susceptibility to *A. tumefaciens*. As the infection with *Agrobacterium* might be considered as a pathogen attack by the plant and the secondary metabolites is known to protect Cannabis against pathogens (McPartland, 1984; Wanas et al., 2016), the difference in susceptibility might be explained by a difference in the secondary metabolite profile (Sorokin et al., 2020). This might partly explain why all stable transformations in Cannabis is done in hemp cultivars (see below; **Table 4**).

**Table 3:** List of *Cannabis sativa* studies reporting transient transformation.

Type of explant	Method	Genotype	Gene of interest	References
Part of leaf Small plants	Agroinfiltration	Unknown	EGL3	Roscow JR., 2017
Cotyledons Leaves	Agroinfiltration	Finola (hemp)	PDS (VIGS) Chl1 (VIGS)	Schachtsiek et al., 2019
Various tissue incl. leaves, male and female flowers	Agroinfiltration	CRS-1; CFX-2; Fedora 17; Felina 32; Ferimon; Futura 75; Santhica 27; Uso31 All hemp	pEarlyGate101-uidA (GUS) PDS (RNAi)	Deguchi et al., 2020
Intact seedlings	Agroinfiltration	Nightingale Green Crack CBD Holy Grail x CD-1 All drug type	pCAMBIA1301 with uidA (GUS)	Sorokin et al., 2020
Leaves	Vacuum infiltration with gold nanoparticles	Tygra (THC low)	GmMYB29A2 GmNAC42-1	Ahmed et al., 2021
Leaves	Protoplasts/PEG	Cherry x Otto II: Sweetened CDB high; THC low	pBeaconGFP_GR-GUS pEVTV_DR5 pBeaconRFP_GUS	Beard et al., 2021
Leaf segments	Agroinfiltration	Cannbio-2 (THC : CBD ratio 1:1.8)	pRNAi-GG-THCAS pRNAi-GG-CBDAS pRNAi-GG-CBCAS pRNAi-GG-CBDAS-UNIVERSAL	Matchett-Oates et al., 2021b
Leaves	Protoplasts/PEG	THC high genotype	GFP	Matchett-Oates et al., 2021a
Leaves	Protoplasts/PEG	Abacus (CBD rich)	CsCBAS : GFP CsCBDAS : GFP CsTHCAS : GFP	Kim et al., 2022
Cotyledons	Protoplasts/PEG	Blueberry Divine (THC : CBD ratio: 20:1) CAN 40 (6:5) CAN 21 (3:100) Charlotte’s Web (85:1) Mantanuska Divine (3:2) Divine (43:100) Lemon Divine (12:5) CAN 20 (89:1) Yunma No. 1 (hemp)	CsMYC2	Zhu et al., 2022

**Table 4:** List of *Cannabis sativa* studies reporting stable transformation.

Type of explant	Method	Genotype	Transformation	Gene of interest	Regeneration	References
Hypocotyl	*Agrobacterium rhizogenes*	CAN0111 (drug) CAN0221 (drug) Futura77 (hemp) Delta105 (hemp) Delta-llosa (hemp)	Stable (Hairy roots)	rol genes	No	Wahby et al., 2013; Wahby et al., 2017
Seedlings	*Agrobacterium tumefaciens*	CAN0111 (drug) CAN0221 (drug) Futura77 (hemp) Delta105 (hemp) Delta-llosa (hemp)	Tumor induction	Wild type *A. tumefaciens*	No	Wahby et al., 2013
Suspension culture based on stem and leaf from seedlings	*A. tumefaciens*	Anka (hemp)	Stable	pNOV3635 containing PMI	No	Feeney and Punja, 2003
Shoot tip	*A. tumefaciens*	Felina 34 (hemp) Fedora 19 (hemp)	Stable	PGIP	Yes	MacKinnon et al., 2001
Hypocotyl	*A. tumefaciens*	Medical Cannabis	Stable	Fluorescent expressed proteins	Yes	Sirkowski, 2012
Hypocotyl Cotyledon Meristem	*A. tumefaciens*	Futura75 (hemp) Ferimon (hemp) USO31 (hemp) USO31 (hemp) USO31 (hemp)	Stable	pBIN19 with uidA (GUS)	Yes, direct regeneration	Galán-Ávila et al., 2021b
Immature embryo hypocotyls	*A. tumefaciens*	DMG278 (hemp)	Stable	pG41sg with CsGRF3-CsDIF chimera and CRISPR/Cas9 guide	Yes	Zhang et al., 2021

Most probably, the recalcitrance in Cannabis explants is also due to the developmental state of the explants. In many plants, the first choice of explant material for transformation would be very young tissue, like that obtained from young seedlings. Such material is easily obtained from hemp, where seeds are available in big amounts. In medical Cannabis, however, very specific combinations of cannabinoids and terpenes might be lost if seeds must be produced. A method where the meristems of medical plants could be the starting material, is therefore highly adventitious. Recent reports show transformation on existing meristems, *in vitro* or *in planta* is possible (Galán-Ávila et al., 2021b; Pandey et al., 2022). Further, developmental regulators can be used to induce new meristems from somatic cells during the transformation process (Maher et al., 2020). One should be aware that the plants obtained from these meristematic techniques might have a higher degree of mosaic tissue than plantlets obtained by regeneration *via* callus.

Within the last six years several papers report transient transformation in Cannabis using not only reporter genes such as GUS or GFP, but also the down regulation of genes using virus induced gene silencing (VIGS) or RNAi (**Table 3**). A study from 2013 (Wahby et al., 2013) showed that both hemp and drug type Cannabis is susceptible to wild type *A. tumefaciens* infection and it is thus not surprising that the most frequently used method for transient transformation is agroinfiltration by vacuum or by using a needleless syringe. A patent by Roscow JR. (2017) describes transient methods to transform Cannabis by vacuum infiltration or by dipping the green parts into an *Agrobacterium* soup where after vacuum is applied. The treated plant parts were producing trichomes comprising secondary compounds on non-flowering parts of the plant. It is, however, unclear if this property was inherited to the next generation. Transient transformation using agroinfiltration is also reported by several others (Schachtsiek et al., 2019; Deguchi et al., 2020; Sorokin et al., 2020; Matchett-Oates et al., 2021b). Results show that the recalcitrance to be transformed might be due to the plants ability to protect itself against pathogen attack, as the addition of ascorbic acid, scavenging excess ROS, had a positive effect of the transformation efficiency (Deguchi et al., 2020). Other methods for transient transformation include vacuum infiltration of DNA coated gold nanoparticles (Ahmed et al., 2021) and transformation of protoplasts using PEG (Beard et al., 2021; Matchett-Oates et al., 2021a; Kim et al., 2022; Zhu et al., 2022).

Transient transformation, although not leading to stably transformed plants, is a very useful tool for overexpression and silencing studies of many genes. Valuable information about the interaction between Cannabis and *Agrobacterium* which might be used to improve stable transformation. It seems that transient transformation is possible in both hemp and drug-type material (Deguchi et al., 2020; Sorokin et al., 2020; Matchett-Oates et al., 2021b), which gives the hope that stable transformation will also be possible in medical Cannabis.

Stable transformation is likely needed for genome editing of Cannabis. The first successful stable transformation of hemp was reported by MacKinnon et al. (2001) in the Scottish Crop Research Institute Annual Report. The report gives very little information about the methods used and the transformation efficiency obtained. Hemp suspension culture cells were transformed by Feeney and Punja in 2003 (Feeney and Punja, 2003). Although showing a transformation rate up to 55%, no plant regeneration was obtained. After these two 20-year-old papers, stable transformation was not reported for almost 15 years, most probably reflecting the lack of success of regenerating transformants.

A patent filed by Sirkowski (2012) describes a method for *Agrobacterium*-mediated transformation of medical Cannabis. The material used is sections of hypocotyl and plants are regenerated from the tissue, but the efficiency is not mentioned. It is not clear in which genotypes the regeneration was successful and if plants were regenerated directly *via* a callus phase.

Very recently, two groups have been able to stably transform hemp (Galán-Ávila et al., 2020; Zhang et al., 2021) (**Table 4**). In the method developed by Galán-Ávila et al. (2021b) shoots are regenerated directly from hypocotyl, cotyledon, or meristems without a callus phase. This transformation system is very fast as tissue could be analyzed for GUS already one month after transformation. Both genotypic and explant differences were observed, with the least genotype dependency and the highest transformation rate found using hypocotyls. This method seems very promising, but the use of hypocotyls might be hampering the transferability to medical Cannabis that is usually propagated using cuttings. However, there was a low transformation rate using meristems as one transformant from one genotype was obtained from this material.

The first paper describing the use of genome editing in Cannabis was published in 2021 (Zhang et al., 2021). Using immature embryo hypocotyls from hemp as a starting material, shoots were regenerated from callus. The phytoene desaturase gene was knocked out using CRISPR/Cas9, giving an albino phenotype. Zhang et al. used a recently developed method, where co-transformation with a GRF-GIF chimeric protein showed a substantially increased efficiency and speed on regeneration, also in recalcitrant genotypes (Debernardi et al., 2020). This was also useful in Cannabis, as overexpression of the developmental regulator chimera CsGRF3-CsGIF1 almost doubled the regeneration rate (Zhang et al., 2021).

## Genome editing in Cannabis - challenges and possibilities

During the last 5-10 years there has been a lot of progress in the prerequisites for genome editing in medical Cannabis. There is now a reference genome for Cannabis and a lot of additional sequence data available. This is a very important basis for the development of sgRNAs for different genome editing techniques. Not only can sequence for candidate genes be found, but also analyses for potential off-targets can be made. The editing techniques are in rapid development with new techniques and improvements emerging every year. The biggest leap forward is the recent reports of regeneration and *Agrobacterium*-mediated transformation of Cannabis. There is, however, still challenges when it comes to using genome editing in medical Cannabis. The two main challenges are genotype dependency and the selection of transformable explant material from cuttings.

As seen in recent experiments, very young hemp tissue can, although maybe not easily, be transformed and regenerated into plants (Galán-Ávila et al., 2021b; Zhang et al., 2021). There is without doubt genotype dependency, not only between chemotypes but also between hemp cultivars. The use of CsGRF3-CsGIF1 in Cannabis and the use of a WUSCHEL family gene in wheat, *T. monococcum*, triticale, barley, and maize are examples where developmental regulators can overcome at least some of the genotype dependency (Zhang et al., 2021; Wang et al., 2022). It is our belief that the future will bring more improvements and refinements as recalcitrance to regeneration and genotype dependency is found in several plant species.

Using hypocotyls from immature embryos for transformation in medical Cannabis is not the obvious choice. The plants are usually clonal propagated to maintain valuable cannabinoid and terpene profiles, which will be changed if plants are propagated by seeds. Recently, promising results showing regeneration from drug type Cannabis using leaf segments and stem nodes have emerged (Önol and Yildirim, 2021; Hesami and Jones, 2021). Methods avoiding young material from seeds also include transformation on existing meristems (Galán-Ávila et al., 2021b) and the induction of new meristems from somatic cells (Maher et al., 2020). All these methods need to be thoroughly investigated in many genotypes of medical Cannabis. The possible outcome of the combination of floral reversion and transformation should also be pursued.

Due to the multiple applications of Cannabis, many traits might be interesting to improve. Single gene traits are easier to work with than very complex traits. However, successful editing of several genes is reported in other species (Gao et al., 2017; Mercx et al., 2017; Morineau et al., 2017) and might also be feasible in medical Cannabis as soon as a versatile transformation platform is established.

Clean cannabinoid products can be obtained by producing them in other systems like yeast (Luo et al., 2019). However, the therapeutic response is often higher using plant products, probably due to a synergistic or entourage effect between various cannabinoids or between cannabinoids and terpenes (Ferber et al., 2020). Genome editing tools would be very useful to study and manipulate the biosynthetic pathways of cannabinoids and terpenes, not only for pure scientific purposes, but also to improve the products in the medical Cannabis industry. Gene editing facilitates gene knockout studies as well as studies altering gene expression levels and tailoring of specific genes. The review by Hesami et al. (2022) lists several ideas for the use of CRISPR-based methods for optimizing cannabinoid production using *in vitro* culture and heterologous systems.

The THCA and CBDA synthases are of special interest, as they are responsible for the synthesis of the two main cannabinoids, THC acid and CBD acid from the common precursor, CBGA. The sequences of these synthases are very similar, and studies have shown that they are positioned at two closely linked loci in a very complex region (Van Bakel et al., 2011; Laverty et al., 2019; Vergara et al., 2019). This complexity makes it challenging, although not impossible, to make changes using genome editing, as guides need to be designed to hit target genes without off-target effects.

As THC is psychoactive, there is a demand for plants completely free of THC. It is essential that drug formulations contain as low a THC content as possible as the THC contamination causes short- and long-term side effects (Volkow et al., 2014). The use of extra purified CBD or synthetic CBD instead of the plant extract results in a lack of other naturally occurring cannabinoids and bioactive compounds such as terpenes involved in the entourage effect. This may have a decisive influence on the therapeutic effect of the product (Russo, 2019). However, Cannabis varieties bred for high CBD content always contain small amounts of THC.

The hemp cultivar Finola has no THCA synthase (Laverty et al., 2019), but still a small amount of THCA is produced (Pavlovic et al., 2019). This presence of THC in CBD-varieties is due to a promiscuous CBDA synthase producing up to 5% THCA (Zirpel et al., 2018). In yeast model systems, site-directed mutagenesis has been performed in THCAS and CBDAS to investigate the importance of amino acids for activity and specificity. One amino acid change at A414V increased the catalytic activity 3.3-fold and caused a shift in specificity profile from CBDA to THCA production (Zirpel et al., 2018). Further studies are needed to uncover the potential of modulating the specificity of this enzyme *in planta*. CRISPR technology would be the obvious choice for inducing base editing in the CBDA synthase and modulate enzyme activity towards making the synthase completely specific for CBDA production.

Another need is to produce plants with a higher content of rare cannabinoids or to change the content of different terpenes independent of the cannabinoid profile. CBG is a compound having medical properties of its own, but it is often present in quite low levels. Knocking out both THCA and CBDA synthases by genome editing would result in a plant with a higher level of CBG.

Genome editing approaches have been used to obtain resistance against plant diseases caused by viruses, fungi, and bacteria (Borrelli et al., 2018), as inactivation of susceptibility genes often gives resistance. This is highly relevant in medical Cannabis production as the loss of production batches due to attack by fungal diseases is a serious problem. The plants cannot be treated with fungicides as traces of these compounds might be found in the final product. Controlling the humidity is highly energy demanding, but if not done, production batches might be lost.

Amino acid changes in the *Mlo* gene(s) are known to give resistance against powdery mildew in a range of plants (Kusch and Panstruga, 2017). In medical Cannabis, where the use of pesticides is not possible, the growth of resistant plants is highly desirable. When medical Cannabis is produced in greenhouses, there is a high humidity, which is the perfect environment for the development of the fungal disease powdery mildew. CRISPR-mediated changes in the *Mlo* gene would give resistance against powdery mildew without any site effects in the cannabinoid production. Pleiotropic effects might occur when the *Mlo* gene is mutated (Jørgensen, 1992). Whereas some *Mlo* mutations seem to be without or with only minor pleiotropic effects, TALEN-derived *mlo* wheat plants with the triple knockout mutations show strong chlorotic symptoms (Jørgensen, 1992; Acevedo-Garcia et al., 2017). This suggests that carefully selected base editing might be preferred over CRISPR/Cas-mediated knockout.

Grey mold due to the fungus *Botrytis cinerea* is a severe problem in indoor Cannabis production. Botrytis is a necrotrophic fungal pathogen, but it apparently starts its infection in a biotrophic manner (Veloso and van Kan, 2018). Rather than indiscriminately killing its host, Botrytis gently guides the host plant towards committing suicide through apoptosis, making the fungus able to colonize and digest the plant tissue. If the spore density is low, the infected plants might be without symptoms. When these asymptomatic plants approach flowering, the fungus might switch to necrotrophic lifestyle causing the host plant to succumb (Veloso and van Kan, 2018). This might be the reason why growers often see a very sudden, severe attack of grey mold. Although the molecular mechanisms are not fully understood, silencing of susceptibility genes has been shown to impede infection (Sun et al., 2017). This and similar research might lead the way for (some) resistance against Botrytis obtained by genome editing.

There is a strong apical dominance in Cannabis (Smýkalová et al., 2019). Varieties with less apical dominance might be easier to regenerate in tissue culture and would therefore be the obvious candidates for transformation. Removal of apical buds or the application of plant growth regulators such as phytohormones change plant architecture, giving lower and more branched plants (Kocjan Ačko et al., 2019; Burgel et al., 2020). These more uniform plants make harvest of inflorescences easier. Although apical bud removal in hemp gives higher seed yield (Kocjan Ačko et al., 2019), it is unclear whether higher cannabinoid yield will be found in plants with a changed architecture due to genetic changes. However, plants treated with phytohormones showed the same or reduced inflorescence dry weight, dependent on genotype, with no change in CBD content (Burgel et al., 2020). Although drug type Cannabis, due to selection, has become shorter and more densely branched, there is a great demand for varieties with a standardized plant type, not least with same height and branching, as such plants are suitable for automation. Thus, genes involved in apical dominance, branching and plant height would be very interesting candidates for genome editing.

As mentioned in the doubled haploid section, haploid inducer lines can be produced using CRISPR/Cas to knock out the centromere-specific histone H3 (CENH3) (Kelliher et al., 2019). This would be a very useful technique to use in Cannabis, where no successful doubled haploid technique is yet available. With a robust transformation platform in Cannabis, haploid inducer lines obtained with this technique is surely possible. Haploid-inducer lines with a hemp genetic background would be useful in medical Cannabis as well, as the haploids will not contain any parental DNA from the haploid-inducer parent. Once established, the haploid-induces lines can carry a CRISPR/Cas cassette that would give edited haploid offspring without the editing machinery (Wang et al., 2019).

There are several other traits of general interest for Cannabis production, also in hemp cultivars. Flowering time, fiber quality, lower phytic acid content of seeds, and soil remediation properties are obvious candidates for investigation *via* genome editing (Shiels et al., 2022).

## Conclusion

The very high genetic diversity in *Cannabis sativa* is a great advantage for conventional breeding. However, conventional breeding through crossing and selection is very time consuming. It requires several rounds of backcrossing and is complicated by the dioecious nature of the plants. Further, the introduction of new traits might compromise the cannabinoid and terpenoid profile of medical Cannabis. Targeted improvements at predetermined positions in the genome by gene editing might mitigate some of these challenges.

Cannabis has traditionally been considered a recalcitrant species, in which techniques like genetic transformation and genome editing were very complicated. However, our review providing insight into recent progresses within tissue culture, genetic transformation, gene editing and necessary sequence information on *Cannabis sativa* indicates that this is not the case anymore. Currently, plant regeneration has been reported not only in hemp but also in a few medical Cannabis cultivars. Regeneration improvements using developmental regulators has facilitated the first report on CRISPR/Cas9 mediated genome editing in hemp. Our study suggests that future efforts could be directed towards development of a robust regeneration protocol for differentiated or meristematic tissue from medical Cannabis and thereby form the basis for future targeted improvements of medical Cannabis.

Genome editing is a great tool for scientific investigations and precision breeding for secondary metabolites such as cannabinoids and terpenes. Expression of genes related to these compounds can be fine-tuned or completely removed by editing promoters and/or genes. Precision breeding further gives the possibilities to add new traits such as disease resistance or changed plant architecture without meddling with a known cannabinoid profile. Conventional methods adding new genetic variation will continue to be a corner stone in breeding but once transformation is a routine tool in Cannabis, there are almost unlimited possibilities to improve a wide range of plant traits. Furthermore, since medical cannabis is often grown under contained facilities, any GM regulatory requirements of genome edited plants will be easier to meet.

## Author contributions

HB-P designed the project. CI wrote the initial manuscript which was carefully revised by HB-P. All authors contributed to the article and approved the submitted version.
